# Exploration of cuprotosis-related genes for predicting prognosis and immunological characteristics in acute myeloid leukaemia based on genome and transcriptome

**DOI:** 10.18632/aging.204864

**Published:** 2023-07-13

**Authors:** Yanhui Wei, Zhaoxu Miao, Xuejun Guo, Songwei Feng

**Affiliations:** 1 School of Medicine, Southeast University, Nanjing, China; 2 Department of Haematology, Puyang Oilfield General Hospital, Puyang, China; 3 Puyang Translational Medicine Engineering and Technology Research Center, Puyang, China; 4 Department of Obstetrics and Gynaecology, Zhongda Hospital, School of Medicine, Southeast University, Nanjing, China

**Keywords:** acute myeloid leukemia, cuprotosis, prognosis, TCGA, transcriptome

## Abstract

Background: Acute myeloid leukemia (AML) is a common hematologic malignancy with a generally unfavorable prognosis. Cuprotosis as a new form of programmed cell death has been shown to play an important role in tumorigenesis and progression; However, the relationship between cuprotosis and the prognosis of AML patients remains unclear.

Methods: Transcriptomic and genomics data, along with clinical information, were obtained from the TCGA and GEO databases. Especially, unsupervised clustering and machining learning were used to identify molecular subtypes and cuprotosis-related risk scores respectively. Kaplan-Meier analysis, univariate and multivariate Cox regression, and Receiver Operator Characteristic curve (ROC) were performed to assess the prognosis based on cuprotosis-related genes (CRGs). Moreover, multiple algorithms were used to evaluate immunological heterogeneity among patients with different risk scores. For *in vitro* analysis, the expression of genes involved in CRGs was detected by Quantitative Reverse Transcription Polymerase (qRT-PCR) in AML patients.

Results: Transcriptomic and genome data indicated the immense heterogeneity in the CRGs landscape of normal and tumor samples. Cuprotosis subtype A and cuprotosis regulatory subtype B in the genomics map and biological characteristics were significantly different from the other groups. Furthermore, these two subtypes had lower risk scores and longer survival times compared to other groups. Cox analyses indicated that risk score was an independent prognostic factor for AML patients. In addition, our risk score could be an indicator of survival outcomes in immunotherapy datasets.

Conclusions: Our study demonstrates the potential of CRGs in guiding the prognosis, treatment, and immunological characteristics of AML patients.

## INTRODUCTION

Acute myeloid leukemia (AML) is a cancerous condition that affects the hematopoietic system and is characterized by abnormal proliferation, hindered differentiation, and diminished apoptosis of stem cells [1–4]. The pathogenesis of AML is rooted in molecular genetics and cytogenetic alterations, which also have an impact on the prognosis and treatment of the disease [5, 6]. Despite recent advancements in immunotherapy, there are notable variations in the prognosis of individual patients, and current prognostic classification systems appear inadequate in providing precise assessments for diverse individuals [7]. Consequently, it is imperative to identify new biomarkers that can accurately predict patient prognosis.

Programmed cell death (PCD) is a natural biological process that plays a crucial role in maintaining the stability of an organism. There are several recognized forms of cell death, including apoptosis, pyroptosis, necrosis, ferroptosis, and cuprotosis [8, 9]. Cuprotosis is another way of PCD induced by metal ions after ferroptosis, discovered by Tsvetkov and his colleagues in 2022 [10]. As a cofactor for essential enzymes, copper ions maintain a low concentration and maintain a dynamic balance [11], and play an important role in the homeostasis of cells, the accumulation and imbalance of intracellular copper ions will be toxic to cells and even induce cell death [12]. The study found that cuprotosis is caused by an excess of intracellular copper transported to mitochondria via ion carriers and directly bound to lipid-acylated components of the tricarboxylic acid cycle during mitochondrial respiration, resulting in the aggregation of lipid-acylated proteins and loss of iron-sulfur cluster proteins, which induces proteotoxic stress and ultimately leads to cell death [10, 13]. Cuprotosis, as a newly discovered form of PCD, holds potential in the diagnosis and treatment of tumors [14], however, the relationship between cuprotosis-related genes and AML (non-M3) prognosis is unclear and is an area of ongoing research.

In this study, we downloaded gene expression data from The Cancer Genome Atlas (TCGA) database for the AML cohort and matched clinical data, and we determined the prognostic value of CRGs for AML. In addition, we also delved into the impact of CRGs on the immune microenvironment of AML patients. Our findings indicate that the CRGs have the potential to provide an effective and precise prediction of the prognosis for AML patients. Importantly, these findings also offer novel insights into the immunotherapy of AML.

## MATERIALS AND METHODS

### Data collection

The TCGA-LAML dataset was downloaded from the TGCA database, and GSE37642 (GSE37642-GPL96, GSE37642-GPL570) and GSE12417 (GSE12417-GPL96, and GSE12417-GPL570) were downloaded from the GEO database. Mutation data and copy number variation (CNV) data were also downloaded from TCGA database. We used the “sva” package to eliminate the batch effect in RNA-seq and microarray, Finally, we annotated 11,1917 protein-coding mRNA in the meta dataset. We excluded incomplete survival information, non-whole bone marrow sequencing, M3, and repeated sequencing samples. Finally, 116 patients were included in the TCGA cohort, 539 patients in the GSE37642 cohort, and 233 patients in the GSE12417 cohort. Cuprotosis-related genes (CRGs) were collected from previous references. The expression data were log2 transformed and normalized. The Gene MANIA database was used to construct protein-protein interactions (PPI) network.

### Unsupervised consensus clustering

In the meta-cohort (combined TCGA dataset and GEO dataset), based on prognostic CRGs, we used unsupervised consistent clustering (“ConsensusClusterPlus” package) to divide all patients and principal component analysis (PCA) was used to determine whether each subtype was relatively independent of the others. Importantly, 1000 repetitions were performed, pltem = 0.8, to verify the stability of the subtype in unsupervised consistent clustering.

### Functional enrichment analysis

Gene set variation analysis (GSVA) was used to evaluate differences in biological pathways between subtypes, using c2.cp.kegg.v7.0.symbol as a reference gene set, FDR < 0.05 was the threshold. Moreover, we used the “limma” package to analyze the differential expression genes (DEGs) between different subtypes, next, we used “clusterProfiler” package for functional enrichment based on DEGs, *P*-value < 0.05 and *q*-value < 0.05 was the threshold.

### Tumor immune microenvironment

Overall, following the pipeline in previous studies, we used eight algorithms (TIMER, CIBERSORT, QUANTISEQ, MCP-counter, XCELL, EPIC, ssGSEA, and ESTIMATE) to estimate the abundances of immune cells for each sample in TCGA and GEO dataset.

### Response to targeted drugs

Relevant references have confirmed the important role of the cell cycle, PI3K/mTOR pathway, and Wnt pathway in the progression of AML. Hence, we used the “pRRophetic” package to calculate the half maximal inhibitory concentration (IC50) of different targeted drugs, including CGP.60474 (cell cycle), JW.7.52.1 (PI3K/mTOR), and CHIR.99021 (Wnt).

### Machine learning-derived risk cuprotosis-related risk score

We performed our previous workflow to construct a consensus prognosis model for AML patients. Firstly, we constructed 55 combination of machine learning algorithms based on nine algorithms, including LASSO, GBM, Survival-SVM, SuperPC, ridge regression, plsRcox, CoxBoost, StepCox, and Enet [15]. We used models that can perform variable filtering as the pre-model. Subsequently, we used the GSE37642 as the training set to construct signatures in prognostic DEGs matrix based on different cuprotosis subtypes. We selected the best consensus prognostic model based on the mean C-index of the three cohorts (GSE37642, TCGA-LAML and GSE12417). Risk score was calculated for each patient based on the expression of each candidate variable and the coefficients in the final model. ROC curve was used to evaluate the predictive performance of risk score for OS. The prognostic difference was analyzed by Kaplan-Meier analysis and log-rank test. In addition, we downloaded the IMvigor-210 cohort, and GSE78220 cohort from the previous references to generate a risk score and verify the effect of the risk score in the immunotherapy cohort.

### Quantitative real-time polymerase chain reaction

Peripheral blood samples were collected from AML patients (12 cases) and healthy volunteers (11 cases) from Puyang Oilfield General Hospital. Patient RNA was extracted by Trizol reagent (Invitrogen, Carlsbad, CA, USA) according to the manufacturer’s protocol and cDNA was synthesized by reverse transcription kits (TAKARA, Japan). QRT-PCR was performed using a Step One Plus qRT-PCR machine (Applied Biosystems, Waltham MA, USA). All primers were provided by Suzhou Jinweizhi Biotechnology Co., Ltd (Jiangsu, China). The primer sequences as shown in Table 1.

**Table 1:** Primer sequences.

**Gene**	**Primer sequences (5′–3′)**
β-actin-F	CACCCTGAAGTACCCCATCG
β-actin-R	GATAGCACAGCCTGGATAGCA
LIPT1-F	TGGATGTGCAGGCTACCAAA
LIPT1-R	CGGCCGATCTTAGAAGCTGT
DBT-F	ACCTGAAGTAGCCATTGGGG
DBT-R	AGCGTGACATTGTAGCACCA
DLST-F	CTGCCTGGGGTCTCCTTATG
DLST-R	AAACGCTGGGGTTTTGACTG

### Statistical analysis and software

The statistical analyses were conducted in the R software (version 4.1.2). Specific statistical methods have been mentioned in the bioinformatics methods above. ^***^, ^**^, ^*^, ns refers to *p* < 0.001, < 0.01, < 0.05, and not significant, respectively. The Microsoft Office PowerPoint software is used to generate images for this article.

## RESULTS

### Genetic variation and expression of cuprotosis-related regulatory genes in AML

**Figure 1:**
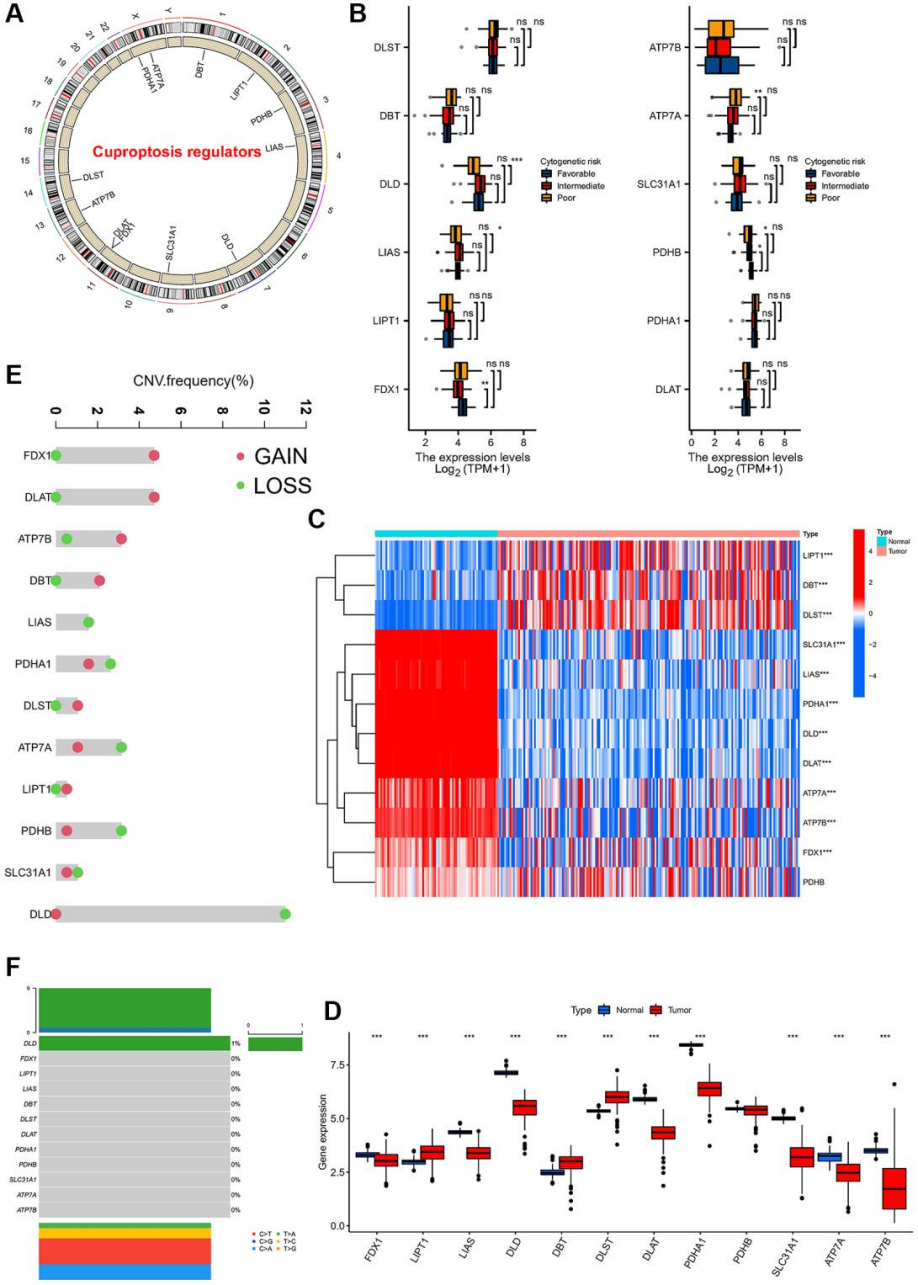
**Expression and variation profiles of 12 cuprotosis-related genes in AML patients.** (**A**) Location of CNV of 12 Cuprotosis genes on chromosomes. (**B**) Expression of 12 cuprotosis-related genes in AML patients with different risk stratification. (**C**) Heat map of cuprotosis-related genes in AML patients and normal population. (**D**) Difference in the expression level of cuprotosis-related genes between normal and tumour samples. (**E**) CNV frequency of Cuprotosis genes, the copy number amplification, green dot; the copy number deletion, red dot. (**F**) Mutation frequency of 12 cuprotosis-related genes in 130 samples. (^*^P < 0.05, ^**^P < 0.01, ^***^P < 0.001).

We first explored the landscape of 12 annotated CRGs in meta cohorts. The location of the 12 CRGs in the chromosomes was shown (Figure 1A). We further compared the expression of these 12 CRGs in different risk categories (ELN 2017 risk category) of AML. Among AML patients in different risk categories, we found differences in DLD, LIAS, FDX1, ATP7A, and PDHD in the ELN 2017 risk category (Figure 1B). Furthermore, we compared the expression of 12 CRGs between normal and AML samples, and the results showed the expression of 11 CRGs (except for PDHB) was significantly different in normal and AML patients (Figure 1C, 1D). In detail, the expression of genes LIPT1, DBT, and DLST were significantly higher in AML patients. We also found CNV amplification in FDX1, DLAT, ATP7B, DBT, DLST and LIPT1 among these 12 CRGs, while mainly showed loss in other CRGs (Figure 1E). We also found DLD mutations with a frequency of only 1% (Figure 1F). The above results showed the expression difference maybe not be caused by somatic mutations, but by CNV.

### Analysis of the characteristics of different cuprotosis subtypes

**Figure 2:**
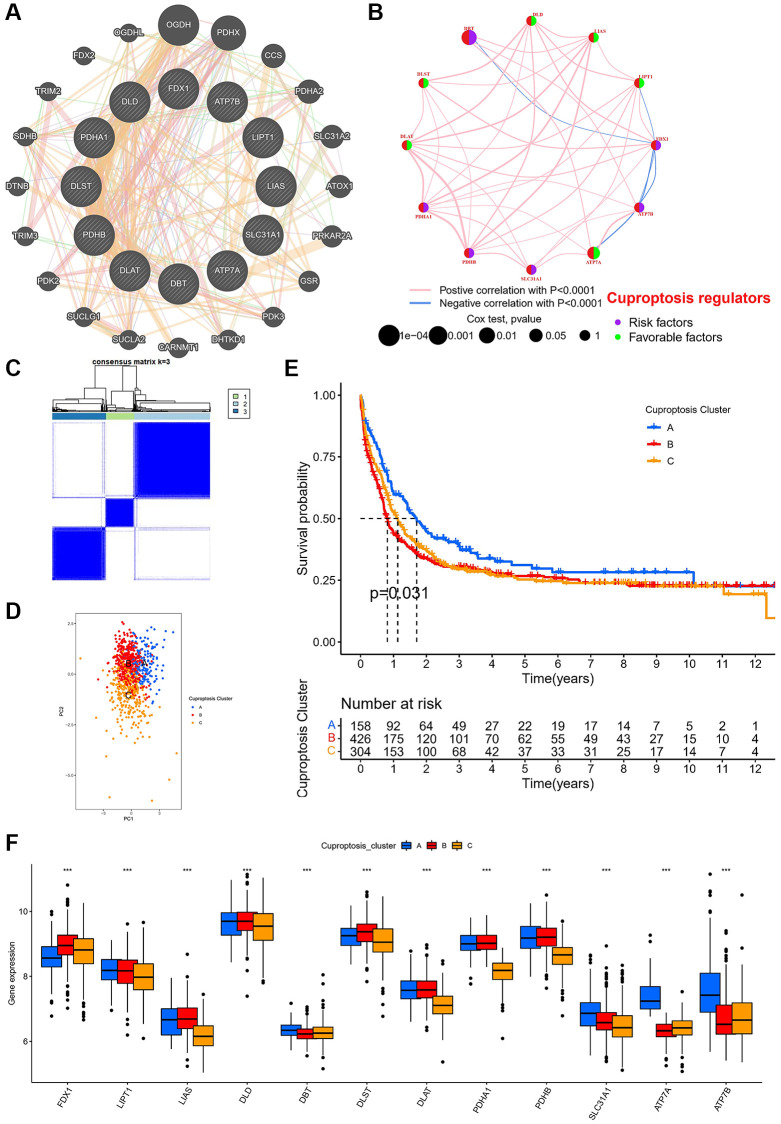
**Cuprotosis modification patterns identified with K-means clustering based on cuprotosis genes.** (**A**) PPI network showing the interactions of the 12 cuprotosis-related genes. (**B**) The interactions of the 12 cuprotosis-related genes in meta cohort. (**C**) Heat map of unsupervised clustering analysis. (**D**) PCA of different cuprotosis subtypes. (**E**) Kaplan-Meier analysis of overall survival for different cuprotosis subtypes. (**F**) Box plot of cuprotosis-related gene expression in different cuprotosis subtypes in AML patients (^*^P < 0.05, ^**^P < 0.01, ^***^P < 0.001).

We investigated the relationships between the 12 CRGs, and the network showed that all 12 CRGs were associated (Figure 2A). Multiple data sets were normalized to increase the sample size (Supplementary Figure 1). In the network of survival and correlation analysis, the results showed that 12 CRGs were still highly associated (Figure 2B). In addition, we investigated the prognostic value of 12 CRGs, we carried out Kaplan-Meier analysis of each CRGs, and the results showed that 11 CRGs had statistical significance on the overall survival (Supplementary Figure 2). Then, we divided AML patients into 3 subtypes, called cuprotosis subtypes A (*n* = 158), B (*n* = 426), and C (*n* = 304) (Figure 2C). The above various subtypes were demonstrated by PCA to be independent of one another over the whole gene expression spectrum (Figure 2D). Patients with subtype A had the best survival compared to other subtypes, according to a Kaplan-Meier analysis (Figure 2E). We further analyzed the expression of 12 CRGs in the three subtypes and all 12 CRGs were significantly differentially expressed in three cuprotosis subtypes (Figure 2F).

**Figure 3:**
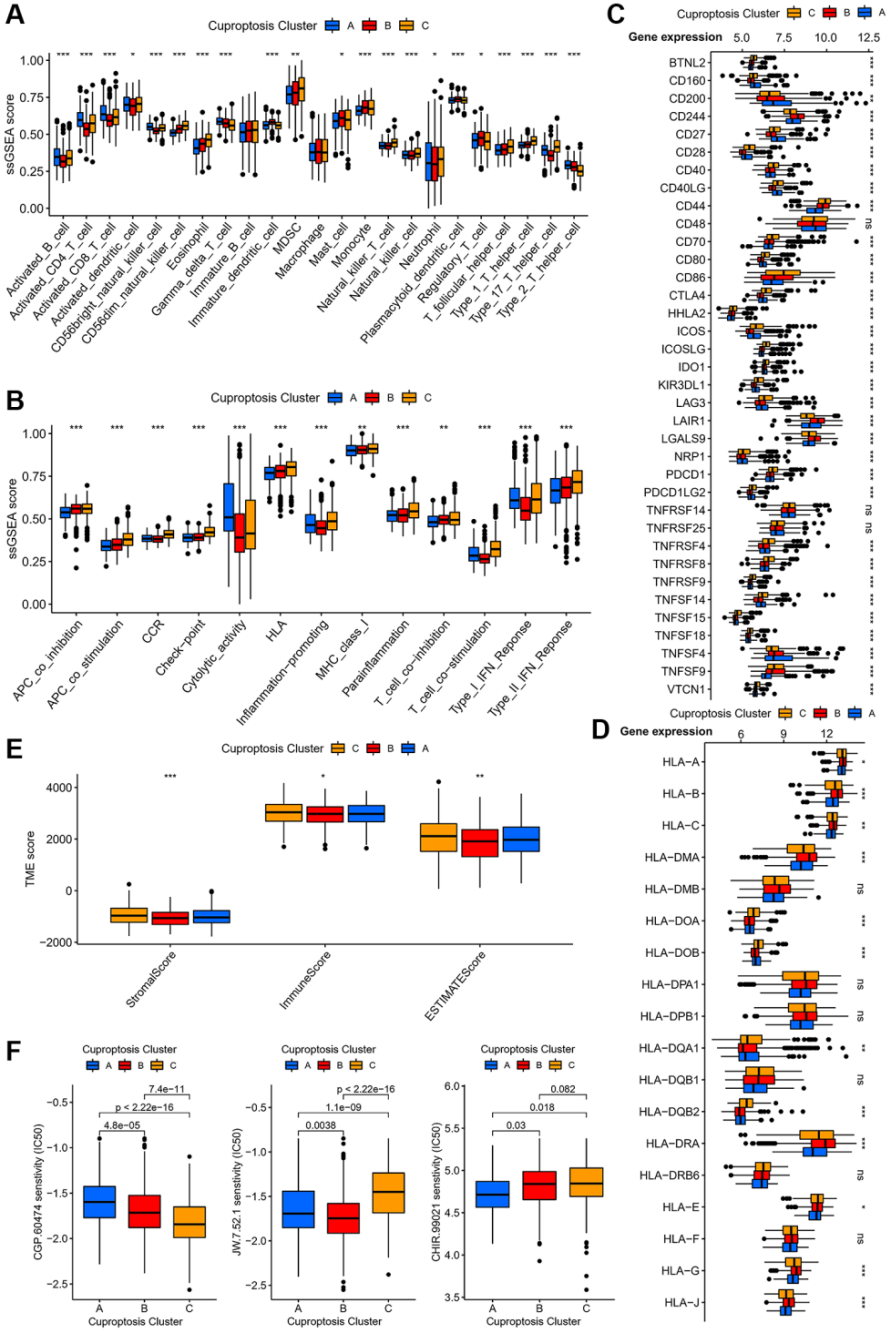
**Immunological and biological characteristics of different cuprotosis subtypes.** (**A**) Expression of immune-infiltrating cells in different cuprotosis subtypes. (**B**) Relationship between immune cell subsets of different cuprotosis subtypes and immune related functions. (**C**) Expression of immune checkpoints of different cuprotosis subtypes. (**D**) Box plots of the expression of different human leukocyte antigens in the three cuprotosis subtypes. (**E**) Analysis of differences in TME scores for three cuprotosis subtypes. (**F**) IC50 of three targeted drugs in different subtypes. (^*^P < 0.05, ^**^P < 0.01, ^***^P < 0.001).

To investigate the immunological characteristics of the three cuprotosis subtypes, we analyzed the relationship between the aforementioned cuprotosis subtypes and immune cells. The results revealed significant differences among the three subtypes in the level of infiltration of most immune cells, with high expression of activated B cells, CD4^+^ T cells, CD8^+^ T cells, dendritic cells, natural killer cells, γδ T cells, neutrophils and type II helper T cells in subtype A (Figure 3A). In addition, the three subtypes of the immune system displayed notable differences in antigen presentation, cytokine receptors, inflammatory response, and T cell activation and suppression (Figure 3B). Additionally, we explored the differences in immune checkpoints among the subtypes and found that there were differences among most immune checkpoints (Figure 3C), with significantly increased expression of CD160, CD200, and CD27 in subtype A. Importantly, we discovered differences among the subtypes in most human leukocyte antigen class I-related gene (Figure 3D). According to the ESTIMATE algorithm-based results, there were disparities in the tumour microenvironment (TME) scores between the three subtypes, with subtype A score lower on both the immune and stromal scores (Figure 3E). Relevant references have confirmed the important role of the cell cycle, PI3K/mTOR pathway, and Wnt pathway in the progression of AML. It’s interesting to note that different subtypes respond differently to specific medications (Figure 3F). Specifically, subtype A was more sensitive to CHIR.99021, subtype B to JW.7.52.1, and subtype C to CGP.60474.

Moreover, we further analyzed the biological functions of different cuprotosis subtypes using GSVA. Enrichment analysis revealed that expression of subtype B was upregulated in the majority of metabolic pathways, including gluconeogenesis, pyruvate metabolism, amino acid metabolism, etc. (Supplementary Figure 3A). Contrarily, subtype A is mainly enriched in the porphyrin and chlorophyll metabolism and cytokinesis pathways (Supplementary Figure 3B), and differences in these pathways may be the reason why subtype A has a higher prognosis for survival when compared to other subtypes. Importantly, almost pathways were enriched in nutrient metabolism and circulation pathways, which was consistent with previous references on the intracellular mechanism of action of CRGs [13].

### Characterization and identification of cuprotosis-regulatory subtypes

We identified 525 common DEGs between each other, and the above genes were enriched in cell cycle and metabolic pathways (Supplementary Figure 3C). Similarly, all samples were divided into 2 subtypes by unsupervised cluster analysis performed by DEGs, cuprotosis regulatory subtype A (*n* = 492) and B (*n* = 394) (Supplementary Figure 3D), and survival analysis of the two regulatory subtypes showed that regulatory subtype B had a better median survival time than regulatory subtype A (Supplementary Figure 3E). Subsequently, we analyzed the expression of 12 CRGs in two regulatory subtypes. In detail, the results showed that FDX1, LIAS, DLD, DLAT, PDHA1, PDHB, ATP7A, and ATP7B were significantly different (Supplementary Figure 3F).

### Construction and validation of a risk score and prognostic model

**Figure 4:**
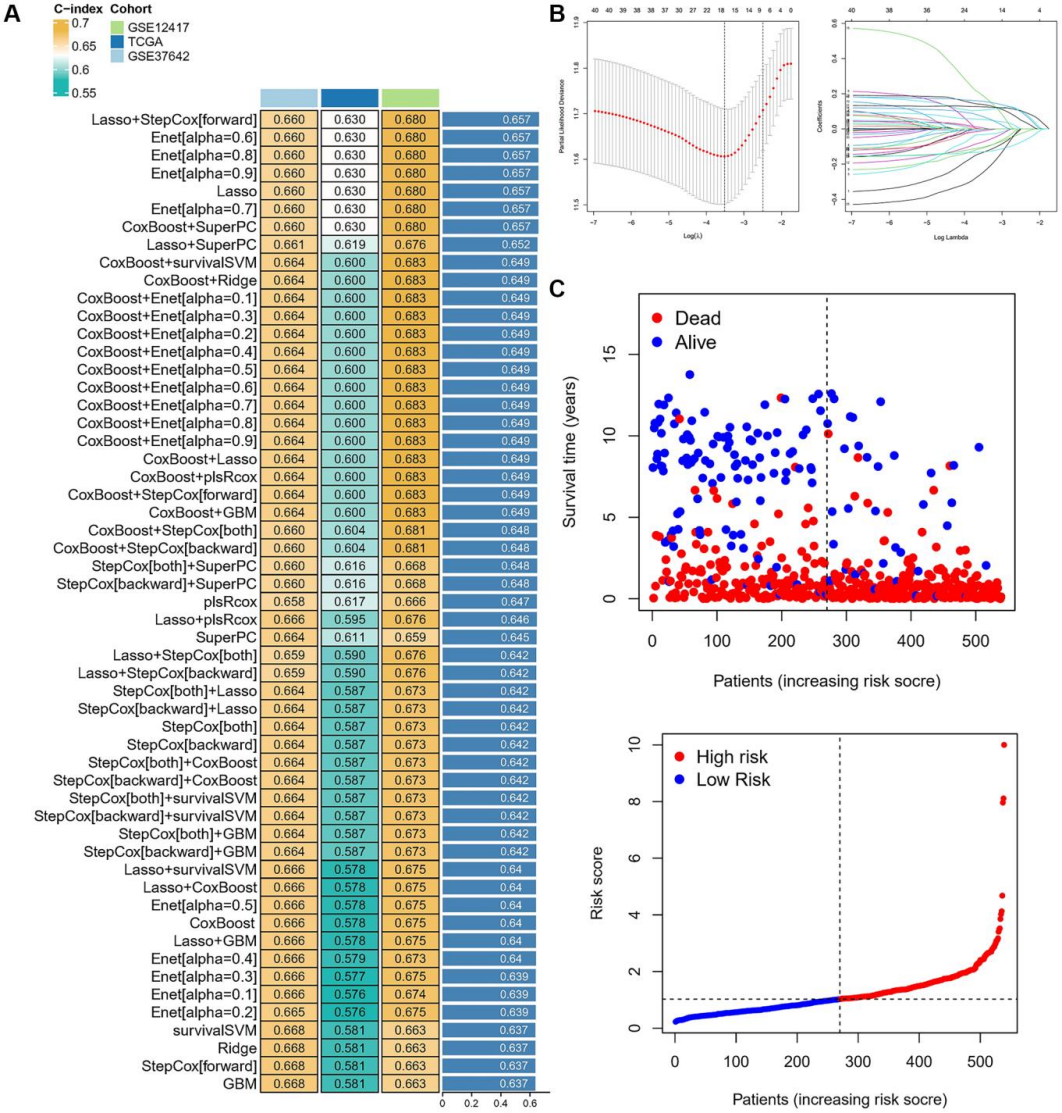
**Characterization of different cuprotosis-regulatory subtypes.** (**A**) The C-indexes of 55 machine-learning algorithm combinations in the three cohorts. (**B**) LASSO coefficient profiles and cross-validation for tuning the parameter selection in the LASSO analysis. (**C**) Risk Score and Survival Status.

**Figure 5:**
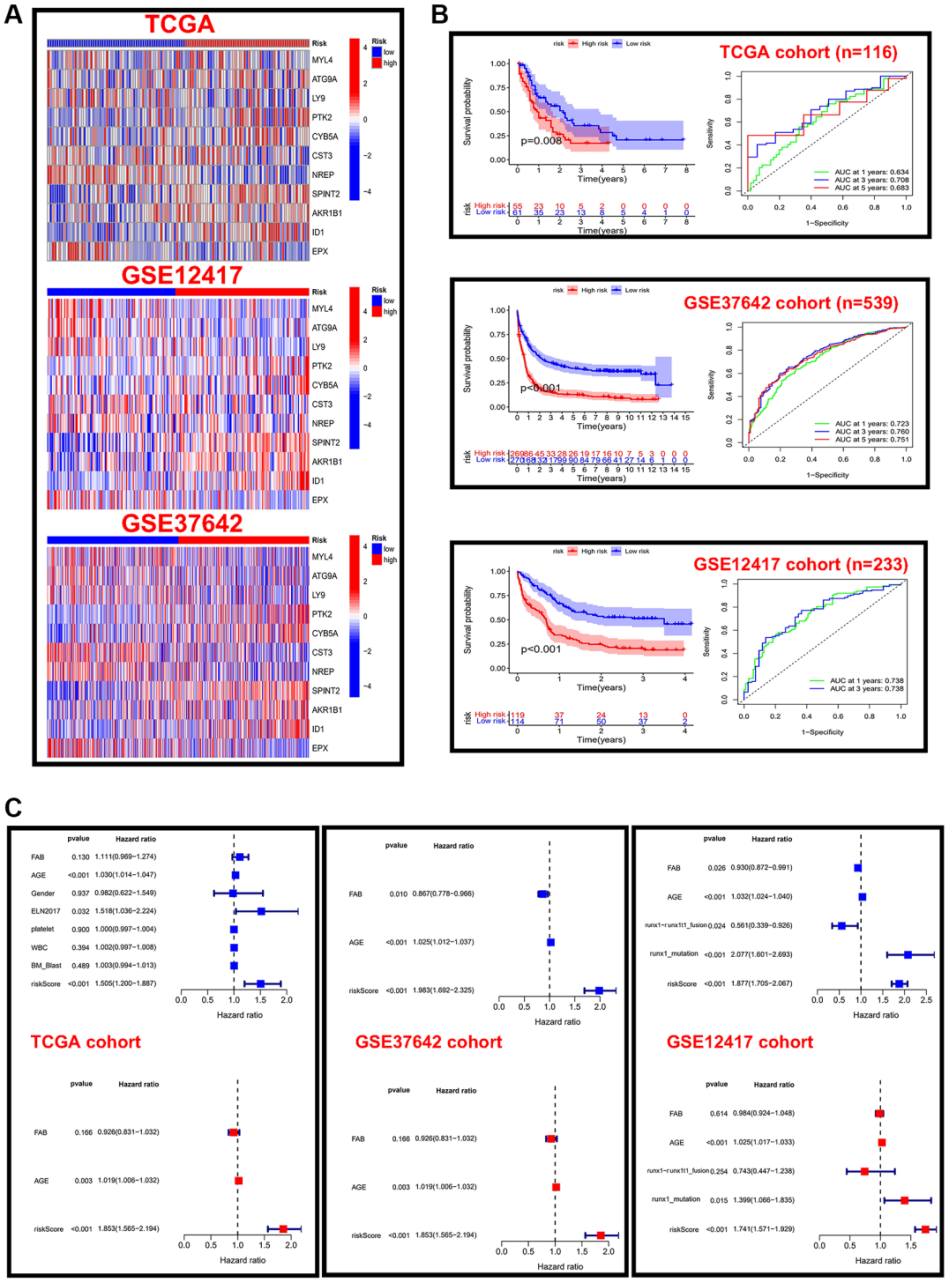
**Validation of risk scores in different cohorts.** (**A**) Heat map of 11 prognostic model genes expressed in different risk score groups in the TCGA and GEO cohorts. (**B**) Kaplan-Meier analysis and time-dependent ROC curve analysis for different risk score groups in the TCGA cohort. (**C**) Forest plots of independent prognostic factors for univariate and multifactorial Cox analysis in three cohorts.

The above construction of different subtypes provides some reference value for the prognosis of AML, but the above two kinds of subtypes only represented population. However, the absence of some clinical characteristics and individual assessments may result in clinical practice not being available. We calculated the C-index in all cohorts and selected the LASSO+Cox (stepwise) algorithm as the final model (Figure 4A). In detail, redundant genes were removed by LASSO regression, and 11 regulators were screened and the coefficients of each regulator were calculated by Cox regression (stepwise) analysis (Figure 4B). Next, the AML sample was divided into low-risk and high-risk groups based on risk scores, and patient fatalities increased as risk scores rose (Figure 4C). We further analyzed the expression of 11 regulators in high-risk and low-risk groups of patients in different datasets revealed that the expression of AKR1B1, ID1, SPINT2, and CYB5A was higher in high-risk patients (Figure 5A). Patients in the high-risk category in various data sets all had significantly shorter OS than those in the low-risk group (*p* < 0.05), according to Kaplan-Meier analysis, which was used to examine the impact of different subtypes on prognosis. The AUC value for the TCGA cohort at 1, 3, and 5 years were: 0.634, 0.708 and 0.683, and the AUC value for the GES37642 at 1, 3, and 5 years were: 0.723, 0.760 and 0.751. For GSE12741 cohort, the AUC value at 1 and 3 years were: 0.738 and 0.738 (Figure 5B). In addition, we again performed univariate and multivariate Cox regression analysis on important clinical features (FAB subtype; age; gender; ELN2017 risk category [16]; leukocytes; platelets; bone marrow blasts; runx1-runx1t1 fusion; runx1 mutation) and risk scores to determine whether they were independent prognostic factors for OS, and the results showed that risk score was an independent prognostic factor (Figure 5C).

To predict the cuprotosis model more accurately at the individual level, we first compared the risk scores of different cuprotosis subtypes and cuprotosis regulatory subtypes and found that cuprotosis subtype A and cuprotosis regulatory subtype B had lower risk scores (Supplementary Figure 4A, 4B), which also corresponded to the better prognostic survival described above. Moreover, we used a Sankey diagram to display the association between cuprotosis subtypes, cuprotosis regulatory subtypes, risk score groups, and prognosis. The results revealed that the majority of the subtypes with bad prognoses were significantly linked to high risk and low survival (Supplementary Figure 4C). Finally, we further explored the composition of immune cells, and the results revealed that the high-risk score group exhibited more Tregs cells, fibroblasts, endothelial cells, and other multiple cell infiltrates (Supplementary Figure 4D, 4E).

### Validation of the risk score in immunotherapy cohorts

**Figure 6:**
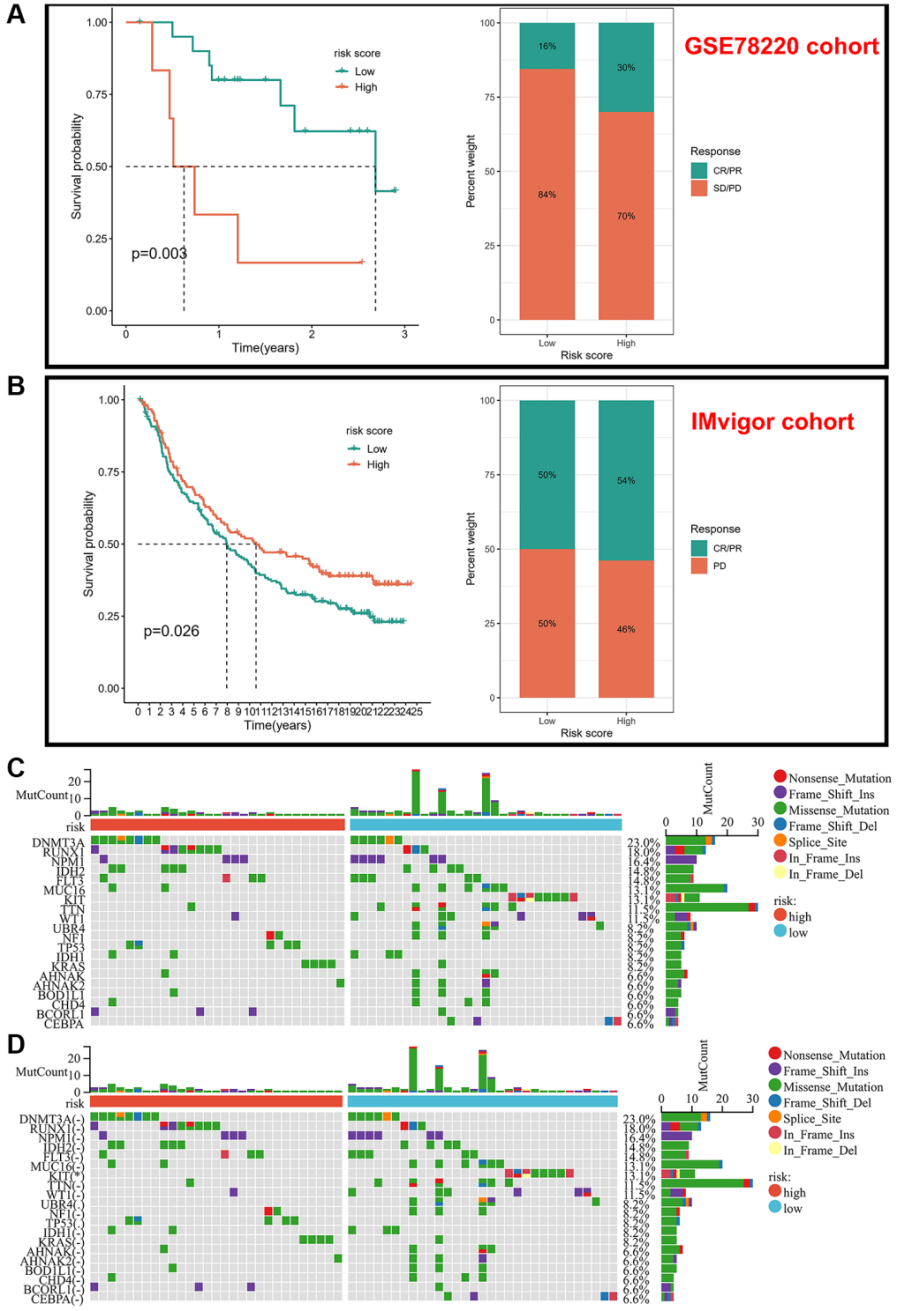
**Validation of risk scores in different immunotherapy cohorts.** (**A**) Kaplan-Meier analysis of different risk scores and response to drugs in the GSE78220 cohort. (**B**) Kaplan-Meier analysis of different risk scores and response to drugs in the IMvigor-210 cohort. (**C**, **D**) Frequency of somatic mutations in different risk score groups.

Due to the lack of information on therapeutic agents for AML patients, we selected immunotherapy cohorts (GSE78220 cohort and IMvigor cohort) as validation sets to predict the response to immunotherapy. In the GSE78220 cohort (anti-PD-L1), we discovered that the low-risk score group had a significantly longer median survival time compared to the high-risk group, while in the IMvigor cohort we found that the survival time and response to treatment was better in the high-risk score group than in the low-risk score group after treating PD-1 treatment (Figure 6A, 6B). Overall, our risk score model can, to some extent, guide the treatment of tumors in anti-PD-1 or anti-PD-L1. It’s interesting to note that prognostic markers for immunotherapy varied based on the various blocking sites. Finally, we analyzed somatic mutations between different risk score subtypes and showed that the frequency of mutations in the KIT gene was higher in the low-risk group, while the frequency of RUNX1 and TP53 mutations was higher in the high-risk group, and these two genes have been included in AML treatment guidelines as molecules with poorer prognosis, but the somatic mutation frequencies were not significantly different between the two groups (Figure 6C, 6D).

### Validation of cuprotosis-related genes in peripheral blood

Considering that DBT, DLST, LIPT1 plays a key role in the cuprotosis-related pathways. Hence, we used qRT-PCR to assess three CRGs in blood samples from 12 AML patients and paired normal patients, the average age of the experimental group was 57.67 ± 14.55-years-old, with a male proportion of 50.00%. The age of the control group was 58.75 ± 10.20-years-old, with a male proportion of 50.00%. There was no statistical difference between the two groups (*P* < 0.05). The results showed that the expression of genes DBT, DLST, LIPT1 was significantly higher in AML patients than in normal patients (Supplementary Figure 5).

### Data availability

Publicly available data sets were analyzed in this study. This data can be found here: Publicly available data sets can be obtained from the TCGA (https://portal.gdc.cancer.gov/), GEO (https://www.ncbi.nlm.nih.gov/gds/).

### Code availability

The code that supports the findings of this study is available from the corresponding author upon request.

## DISCUSSION

In recent years, technological advancements in detection, such as next-generation sequencing, have enabled the development of novel and effective treatments for acute myeloid leukemia (AML), including immunotherapy and molecularly targeted drug therapy. However, as one of the common malignant tumors in the blood system [17], despite these advances, AML remains a major challenge for healthcare systems, with its high heterogeneity resulting in low 5-year survival rates, particularly for young patients (35%) [18], and even poorer prognosis for elderly patients aged 65–74 years [19]. Given these challenges, identifying new prognosis-related genes and developing innovative prognostic models are crucial for the stratification of treatment and prognosis of AML patients.

In recent years, the discovery of cuprotosis as a metal-dependent mode of cell death has garnered significant attention in the field of cancer research [10]. This study analyzed the genetic and clinical characteristics of Acute Myeloid Leukemia (AML) patients in the TCGA and GEO datasets, and found that 11 out of the 12 candidate risk genes (CRGs) were differentially expressed between normal patients and AML patients. Further investigation into the prognostic implications of the expression of the 11 genes revealed a correlation between high expression levels of ATP7B, DBT, FDX1, PDHA1, and PDHB and poor prognosis in AML patients. The ATP7B gene, located on human chromosome 13, encodes the production of the copper-transporting P-type ATPase [20]. Elevated expression levels of ATP7B have been shown to negatively impact chemotherapeutic efficacy in various solid tumors [21–24]. FDX1, a mitochondrial metabolic gene, has a crucial role in various processes, including amino acid and sugar metabolism. Abnormal expression of FDX1 can significantly alter cellular behavior, though its specific impact on tumors has yet to be fully understood [25]. The PDHB gene is a key component involved in gluconeogenesis, and previous studies have demonstrated that high expression levels in lung cancer patients are associated with reduced overall survival [26], which is consistent with our findings. The abnormal expression of the other genes analyzed in this study also holds prognostic significance for AML patients.

In our analysis, we classified patients into three distinct cuprotosis subtypes based on the expression levels of the 12 CRGs. Cluster analysis revealed significant differences in the characteristics, biological behavior, TME immune cell infiltration, and patient prognosis among the subtypes. It was observed that the prognosis of subtype A was more favorable compared to the other two subtypes. Further analysis indicated that subtype A exhibited higher cytolytic activity (CYT) and infiltration of multiple immune cells, including activated B cells, CD4^+^ T cells, CD8^+^ T cells, and natural killer cells. CYT serves as a measure of inflammation and previous studies have shown that high CYT levels in tumors are associated with better overall survival rates [27]. Another study has previously suggested that immune cells CYT could serve as an indicator for the efficacy of immune checkpoint inhibitor therapy in prostate cancer patients. Furthermore, this study determined a correlation between immune cell CYT and overall immune function, with a notable increase in the proportion of CD8+ T cells in the group with high CYT expression as compared to the group with low CYT expression [28]. This result aligns with our findings.

We employed a comprehensive analysis, utilizing 55 combinations of machine learning algorithms to build models in various datasets, and differentiated high-risk and low-risk subtypes based on the results of these models. Our findings indicate that there are significant differences between the two subtypes, which were validated in multiple cohorts, including cuprotosis subtypes and cuprotosis regulatory subtypes, and in certain solid tumors. The lower risk scores for both cuprotosis subtype A and cuprotosis regulatory subtype B may also account for the better prognosis in these two groups. In addition, after incorporating more important clinical characteristics, univariate and multifactor COX regression analyses still showed that our risk score model could be used as an independent factor for prognosis, fully illustrating the stability of the model. Additionally, our analysis of the biological behavior of the different risk subtypes revealed that the high-risk score group exhibited higher infiltration of Tregs cells, endothelial cells, and macrophages. This observation may be associated with a poor prognosis. As a type of immunosuppressive cells, high expression of Tregs cells has been shown to result in the escape of tumor cells [29, 30]. Studies have reported that the expression of Tregs cells is higher in acute myeloid leukemia (AML) patients compared to healthy individuals [31], and that Tregs contribute to immune escape through the suppression of anti-leukemia treatment [32]. Macrophages, as a component of immune cells, play a crucial role in the inflammatory and tumor microenvironment [33], and are often implicated in the establishment of a microenvironment that is favorable to tumor progression through various mechanisms [34]. Tumor-associated macrophages promote tumorigenesis and progression by expressing pro-inflammatory cytokines in an inappropriate manner through disrupted inflammatory signaling pathways [35], and this inflammatory state also contributes to the progression of chronic lymphocytic leukemia (CLL) and the extramedullary infiltration of T acute lymphocytic leukemia (T-ALL) [36, 37]. Importantly, immunotherapy is a widely researched area in cancer treatment and is gaining prominence for improving the cure rate and long-term survival of patients. This includes therapies such as PD-1 antibody and chimeric antigen receptor T cell therapy [38–41]. Our risk score model demonstrated significant differences across the groups receiving PD-1 or PD-L1 therapy, implying that our model holds potential in guiding the application of immunotherapy.

There are some limitations to our research. We only utilized public databases for initial exploration and did not examine real-world data, there may be differences in geography, skin color, age, and other aspects. Additionally, we lack sufficient information about immunotherapy for AML patients, which will be the focus of our future efforts.

In conclusion, as a preliminary study of cuprotosis in AML, we demonstrated the relationship between cuprotosis-related genes, cuprotosis subtypes and prognosis of AML patients. Our study well demonstrates the prognostic value of cuprotosis in AML patients and will inform new AML therapeutic targets.

## Supplementary Materials

Supplementary Figures
