# Supplementary material for: Exploration of cuprotosis-related genes for predicting prognosis and immunological characteristics in acute myeloid leukaemia based on genome and transcriptome
Source: Aging (Albany NY). 2023 Jul 13;15(13):6467–86. doi: 10.18632/aging.204864 (PMC10373958; doi:10.18632/aging.204864)
Supplement: Supplementary Figures [file aging-15-204864-s001.pdf]

SUPPLEMENTARY FIGURES

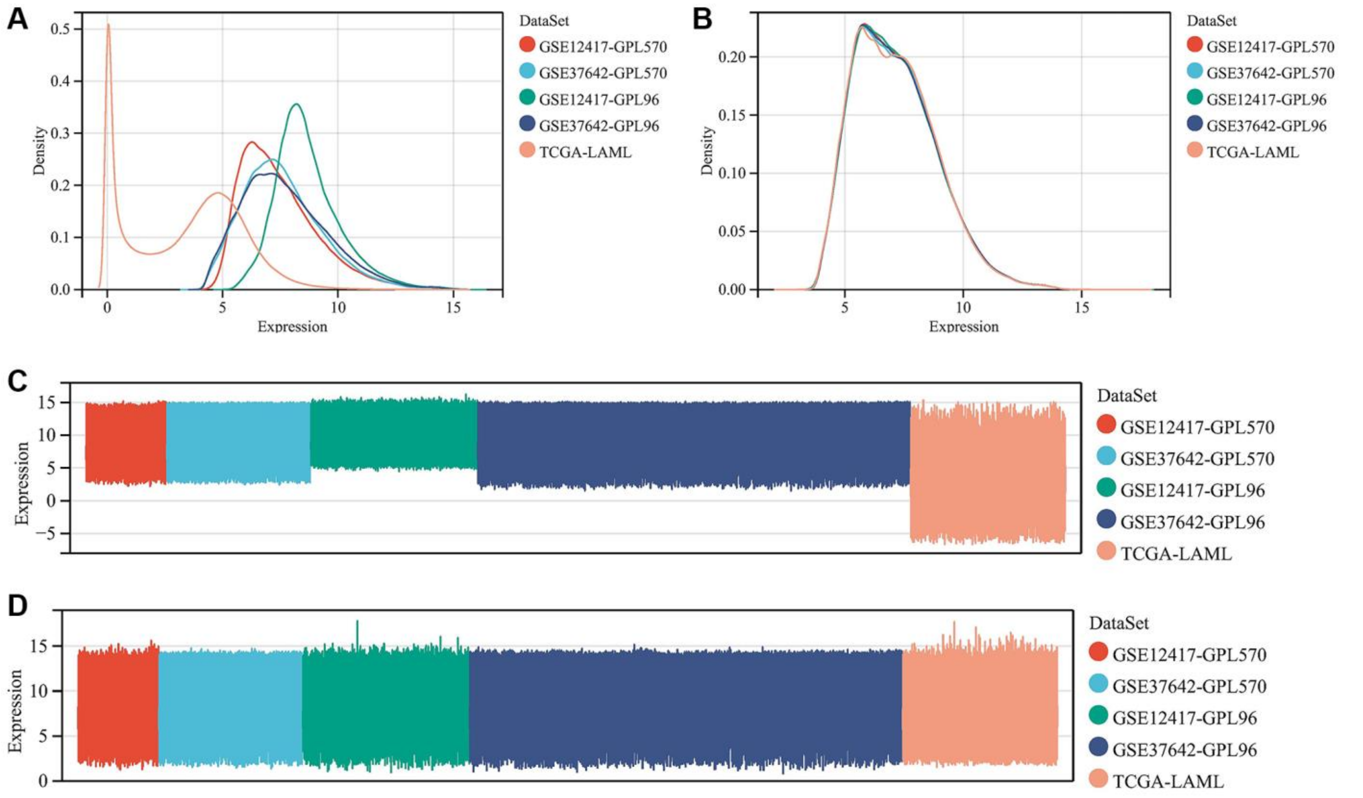

Supplementary Figure 1. Normalization of multiple cohorts. (**A**, **C**) Data characteristics before normalization of the five cohorts. (**B**, **D**) Data characteristics after normalization of the five cohorts.

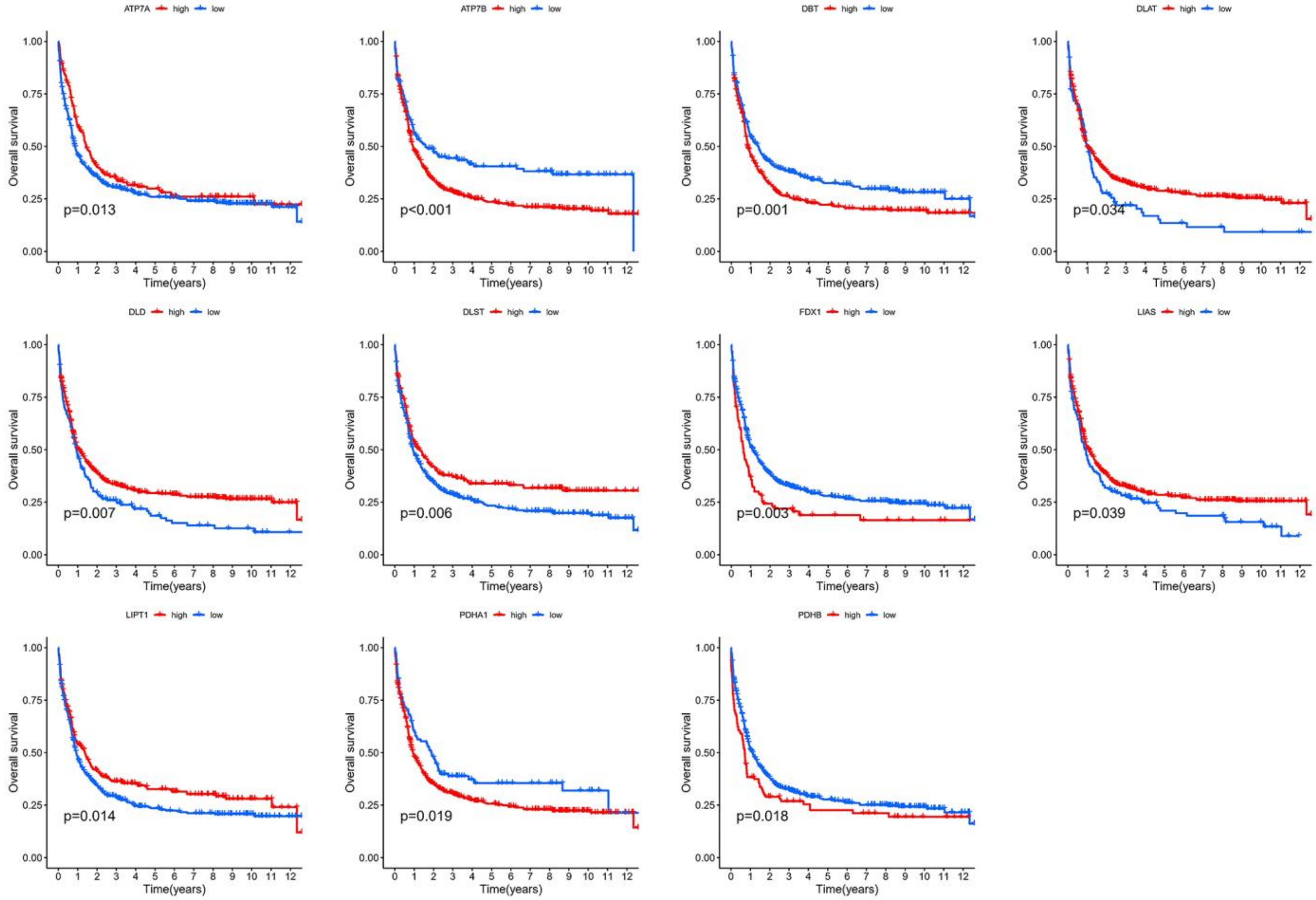

Supplementary Figure 2. Kaplan-Meier analysis of 11 cuprotosis-related genes.

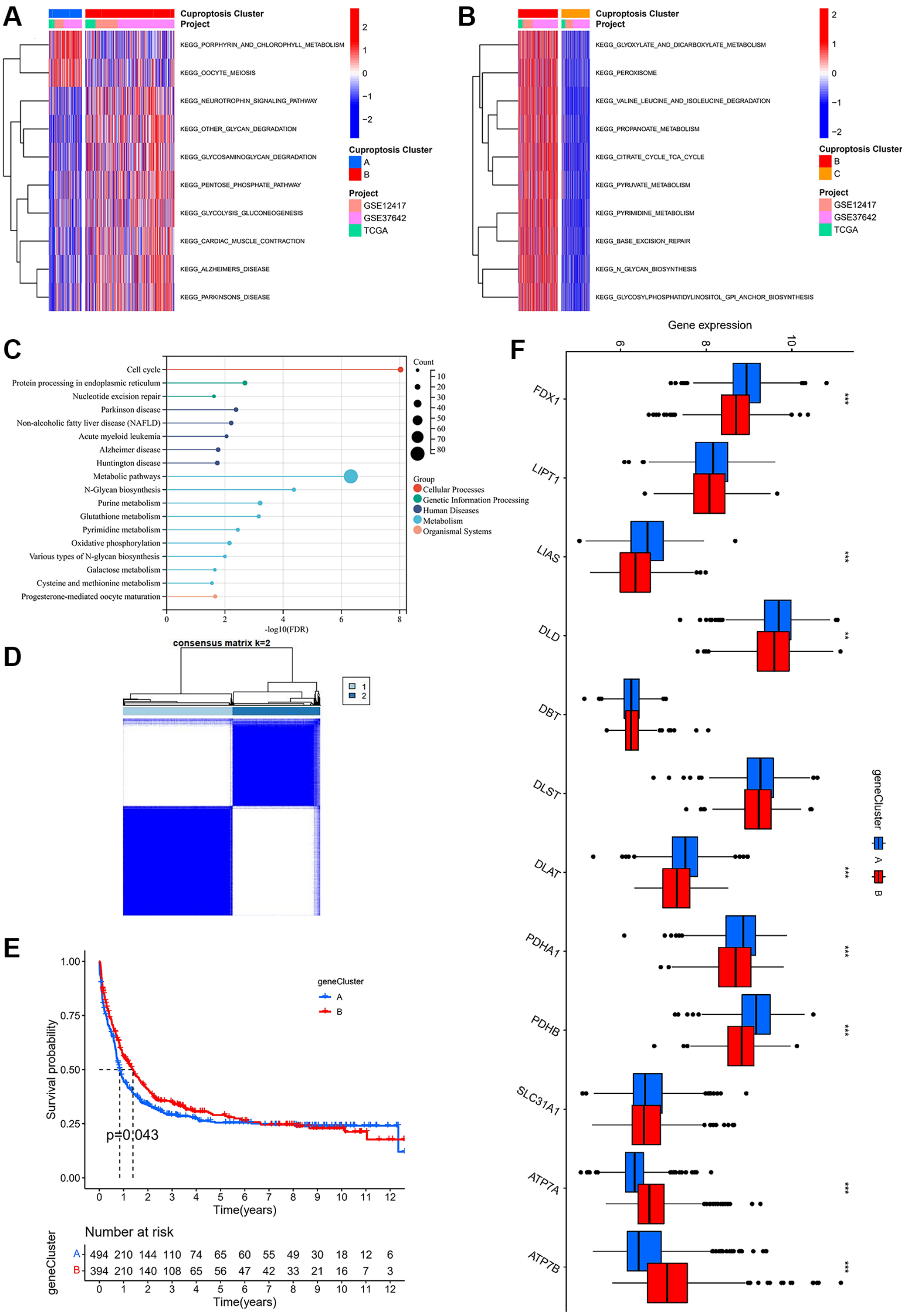

Supplementary Figure 3. Characterization of different cuprotosis-regulatory subtypes. (**A**) Heat map showing KEGG enrichment analysis of cuprotosis subtypes **A** and **B**. (**B**) Heat map showing KEGG enrichment analysis of cuprotosis subtypes **B** and **C**. (**C**) Functional enrichment analysis of DEGs. (**D**) Heat map of unsupervised clustering analysis. (**E**) Kaplan-Meier analysis of overall survival curves for different cuprotosis-regulatory subtypes. (**F**) Box plot of cuprotosis-related genes expression in different cuprotosis-regulatory subtypes ($^{*}P < 0.05$, $^{**}P < 0.01$, $^{***}P < 0.001$).

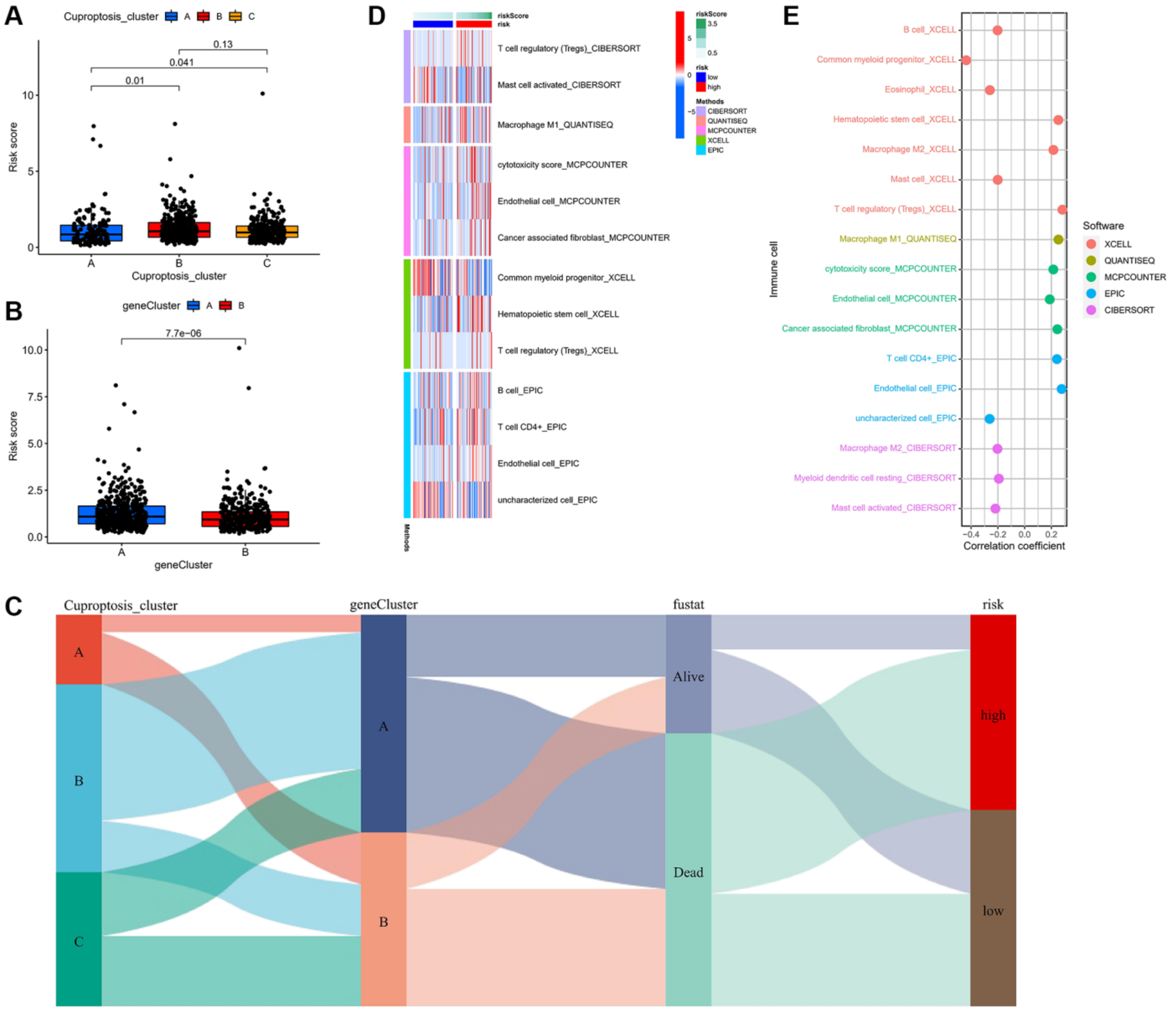

Supplementary Figure 4. TME characteristics of risk score and their relevance with molecular subtypes. (**A**) Risk scores for different cuprotosis subtypes. (**B**) Risk scores for different cuprotosis-regulatory subtypes. (**C**) The relevance in cuprotosis subtypes, cuprotosis-regulatory subtypes, risk score, and survival status. (**D**) Heat map showing the relationship between different risk score groups and immune cells. (**E**) Bubble plots showing the relationship between different risk score groups and immune cells.

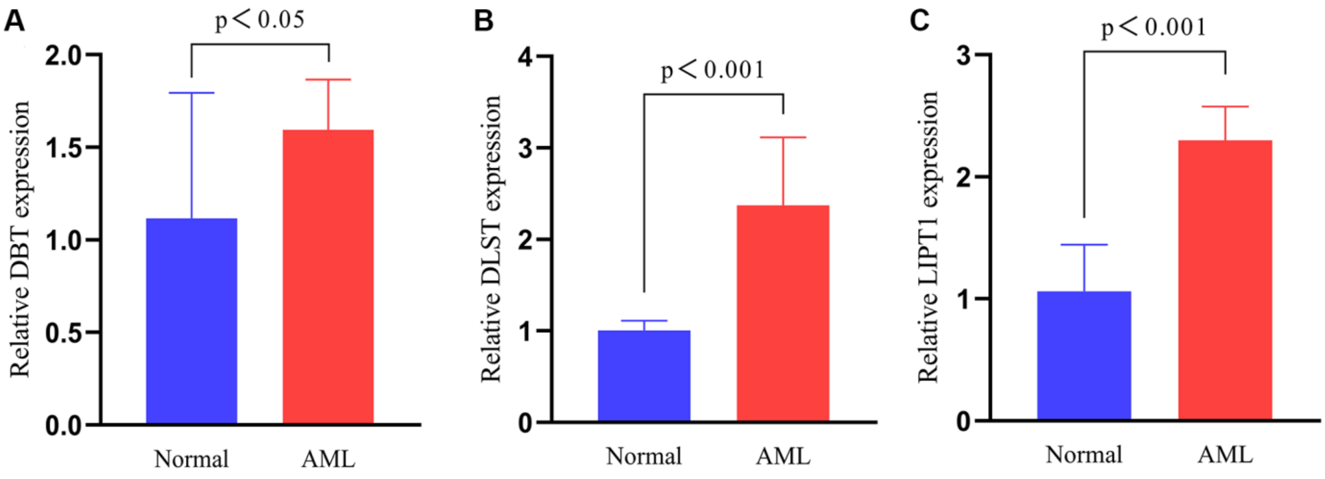

Supplementary Figure 5. Expression of three cuprotosis-related genes in clinical samples. (**A**) Expression of DBT gene in normal human and AML patients. (**B**) Expression of DLST gene in normal human and AML patients. (**C**) Expression of LIPT1 gene in normal human and AML patients.
